# Porcelain as a Filling Material

**Published:** 1905-12-15

**Authors:** Henry C. Raymond


### PORCELAIN AS A FILLING MATERIAL.

BY HENRY C. RAYMOND, D.D.S.

Read before the Michigan State Dental Association, July 12, 1905.

It will be the purpose of this very brief paper to try and enumerate the advantages that porcelain possess over gold and amalgam as a filling material.

The three principal features, or I might say, advantages that porcelain has over gold are, 1st, its quality as a preservative of the teeth; and, 2nd, its application is effected with much less exhaustion to both patient and operator, and 3d, its almost ideal harmonious effect in matching the natural teeth.

The term compatability has been used by one of the most earnest and successful operators in porcelain, when accounting for the superior qualities it seems to have over other materials in preserving the teeth, and though the term has been questioned by some as being incorrectly applied, I know of no other word that so aptly fills the bill. Porcelain seems to more closely resemble tooth substance than anything we have. Its glazed surface is the best substitute for the enamel. In texture, in color, and in resistance to the thermal changes it is more like the natural teeth than is anything else. It does not tarnish as do the metals, and when a perfectly adapted porcelain filling is cemented into a cavity, it forms a more homogeneous addition to the tooth than any metal filling, however perfectly it may be inserted. These features, which porcelain possesses in a marked degree more than any other material, do seem to sum up a compatability with healthy tooth substance and the rurrounding tissues, and for want of a better term, is used to account for its success in arresting dental caries.

A leaking porcelain filling, unless loose, is an impossibility, something which can not be said of gold or amalgam. We all know how difficult it is to put in a water-tight gold filling. Examine all the gold fillings you desire and see how few are free from discoloration at the margins and of the surrounding tooth structure, due to moisture and decomposition beneath the filling. Even many of the gold fillings put in by the most able operators are not free from that dark line surrounding the filling, and in many instances extending well beneath it. This defect is what makes many gold fillings so unsightly in the anterior teeth, presenting as they do the yellow metal, and the darkened enamel and dentine adjacent. It is hardly necessary to add that such fillings are not saving the teeth. We frequently see teeth with large gold fillings in which the enamel is transversed by one or more cracks or checks, which were not present before the filling was put in. In some instances the cracks are caused by excessive malleting, but frequently they are caused by the expansion of the metal against the unyielding enamel; large amalgam fillings will bring about the same result when put in without a cavity lining. It cannot be wondered at that such fillings prove failures in a comparatively short time. Nothing could be more incompatible with tooth structure than the metals, and the wonder is that they have saved as many teeth as well as they have. Sound reasoning and good sense should demonstrate that porcelain is an infinitely better material for the preservation of the teeth, and experience has proved that such deductions are not only logical but are actually clinical facts.

How great a strain is the insertion of a large gold filling on the vitality of the operator, and how exhausting to the average patient, it is hardly necessary to mention, as many patients and operators know too well. While it may take longer to make and insert some porcelain fillings than would a gold filling, many do not take any longer than gold would for the same cavity, and in any case, it is much easier for both patient and operator, as the rubber dam is rarely required and much of the work is done out of the mouth. When using porcelain we are using a material that fulfills more than anything else the requirements for a permanent operation and one that is harmonious with nature in effect, and with a minimum physical demand on both patient and operator. That I think is almost ideal dentistry. Many teeth under gold fillings and some under amalgam are so annoying from thermal changes that the metal has to be removed and replaced with something of a less irritating nature. In many instances the pulp must be devitalized before comfort is restored. Dead pulps with their attendant evils under metal fillings are a common occurence, especially in the anterior teeth, where the protection of the pulp by the dentine is only slight. This rarely occurs with porcelain. In fact, when a metal filling has been removed, owing to the discomfort caused by the irritation from thermal changes, and porcelain is used, in its place, the operation is usually followed with complete comfort, and the pulp is preserved. There are many teeth that cannot be saved with a metal filling, which would have to be crowned, with of course the loss of the pulp. Such teeth may be frequently saved for many years with porcelain, and the pulps preserved in a healthy condition. The average operator is seldom sure that his gold operation is a perfect one, and there are many defects that he is not aware of because he cannot see them. When a porcelain filling is ready for the cavity it can be seen at a glance whether it is defective or not, and if necessary, another can be made, thus ensuring an accuracy of the operation that is very satisfactory. The use of porcelain has been objected to by some on the ground that it is so difficult to manipulate, but I think that the man who can make a gold filling, can, if he will take the time to master the technique of the work, make an equally good porcelain filling. Even the average operator with the right instruction and ground work will succeed in making a creditable operation in porcelain, and in either instance he will make a more beautiful and permanent operation. A good porcelain filling will save a tooth better than the best gold filling that can be made, and a poor porcelain filling will preserve the tooth much longer than would a poor gold filling.

Some writers have asserted that while porcelain is an ideal filling under certain circumstances its field is decidedly limited. I cannot help repeating here a statement I made some time ago, though a writer about a year ago dubbed it as absurd: "The application of porcelain as a filling material is only limited by the ability of the operator."

There are very few places in the mouth where it cannot be used successfully if the operator has thoroughly mastered his work, in fact, I know of no material that can be so extensively used with such satisfying results. Objection has been made that in many locations the margins break away, and that in many instances the cement dissolves out and leaves a space for food and moisture to gather and decompose. When the margins break from porcelain, it is usually the result of faulty occlusion, or, owing to wrong method in cavity preparation, a frail, weak margin results in the failure. With an inlay perfectly adapted to all portions of the cavity, and its margins, it is impossible for enough cement to dissolve out to permit space sufficient for anything to lodge that will undergo detrimental decomposition. The fine line of cement seen when the filling is set does wash or dissolve out, but only to a certain depth, when the space becomes so narrowed that moisture cannot penetrate any farther.

I have rarely seen decay around a porcelain filling, though I have seen many poor ones, and some that have been in nearly twenty years, and some even that have had the margins badly broken away for years. Can the same be said of any other material?

I have said little of the beautiful, natural appearance of porcelain and the difference it makes in the expression of the mouth and face as compared with gold. Even if it were not so good a preserver of the teeth as it is, it would still have a strong claim to its place in the anterior teeth, and very many would rather have it even if it had to be renewed quite frequently. It is almost debasing to a profession, the members of which are supposed to be refined and esthetic, to disfigure and mar the face with glaring yellow metal as it has been done, and is still being done to a great extent.

I have said little of amalgam, as I hardly think it can be considered in connection with porcelain when all the advantages of the latter are summed up. The only advantage that amalgam possesses over porcelain is its ease of manipulation, which enables us to use it in some cavities that it would be almost impossible to fill with any thing else, owing to their in-accessibility. Though I am an ardent advocate for the use of porcelain, it must not be understood from what I have said that I do not think that other materials have any place in our work. The most successful dentist is the one who avails himself of whatever will bring the best results in a given case, always providing that he is thoroughly conscientious in his deductions, and places first and last the patient's best interest above everything else. Such is the faith I have in porcelain as a tooth preserver and because its effects are so in harmony with nature, that were I confined to but one material for saving the teeth, porcelain would be that material.

---

